# *Giardia duodenalis* Induces Apoptosis in Intestinal Epithelial Cells via Reactive Oxygen Species-Mediated Mitochondrial Pathway In Vitro

**DOI:** 10.3390/pathogens9090693

**Published:** 2020-08-23

**Authors:** Lin Liu, Rui Fang, Ziyan Wei, Jingxue Wu, Xiaoyun Li, Wei Li

**Affiliations:** Heilongjiang Key Laboratory for Zoonosis, College of Veterinary Medicine, Northeast Agricultural University, Harbin 150030, China; liulinneau@163.com (L.L.); f15546687372@163.com (R.F.); xiaoyanpaopao@163.com (Z.W.); wujingxue00@163.com (J.W.); lixiaoyun1974@aliyun.com (X.L.)

**Keywords:** *Giardia duodenalis*, Caco-2 cell, ROS, mitochondria damage, caspase, apoptosis

## Abstract

The intestinal protozoan parasite, *Giardia duodenalis*, infects a large number of people in the world annually. *Giardia* infection has been considered a negative effect on intestinal epithelial cell growth, while the underlying mechanisms remain to be explored. Here we evaluated reactive oxygen species (ROS) production and apoptotic events in *Giardia* trophozoites-stimulated Caco-2 cells via fluorescence microscopy, transmission electron microscopy, flow cytometry, western blot, and cell counting kit-8 analyses. The results showed that *Giardia* trophozoite treatment could induce lactate dehydrogenase release and Caco-2 cell apoptosis. The ROS levels were increased post treatment. The observed typical characteristics of mitochondria damage include significant swelling and degeneration of matrix and cristae. After trophozoite treatment, the level of Bax protein expression was increased, while Bcl-2 protein decreased. Trophozoite stimulation also led to reduction of mitochondrial membrane potential and release of cytochrome c from the mitochondria to the cytoplasm, and this process was accompanied by activation of caspase-9 and caspase-3 and poly (ADP-ribose) polymerase 1 cleavage. Pretreatment with N-acetyl-L-cysteine, a ROS inhibitor, reversed *G. duodenalis*-induced Caco-2 cell apoptosis. Taken together, we indicated that *G. duodenalis* could induce Caco-2 cell apoptosis through a ROS- and mitochondria-mediated caspase-dependent pathway. This study furthers our understanding of the cellular mechanism of the interaction between *Giardia* trophozoites and host cells.

## 1. Introduction

The protozoan parasite *Giardia duodenalis* affects nearly 280 million people per year. In 2004, the World Health Organization classed giardiasis as a neglected tropical disease [1]. The life cycle of *G. duodenalis* involves two major stages: infectious cysts and disease-causing trophozoites. *Giardia* infection begins with the ingestion of cysts by susceptible hosts through contaminated food and water and release of trophozoites from the cysts under the stimulation of the gastric acid and bile. Then trophozoites move towards the small intestine and attach tightly to intestinal epithelial cells (IECs) via the ventral disc [2]. The direct interaction between *Giardia* trophozoites and IECs increases permeability of the intestinal barrier through inducing cellular damage, disrupting epithelial tight junctions, and compromising brush border enzyme function [3], leading to malabsorption, maldigestion, electrolyte imbalance, and ultimately diarrhea of infected individuals [4]. Many individuals infected with *Giardia* develop acute intestinal and extra-intestinal disease, while some are asymptomatic; the pathogenesis of *Giardia* remains obscure despite extensive investigations [5].

The interaction between host cells and *Giardia* trophozoites is a primary initiator of disease development [1]. The Caco-2 cell line, a validated in vitro human IEC model derived from colon carcinoma, shows similar morphology and functions to the mature enterocyte [6,7]. Transcriptome analysis revealed that *Giardia* trophozoites could induce an up-regulation of various chemokines (CXCL1, CXCL2, CXCL3, CCL2, and CCL20) and apoptosis in differentiated Caco-2 cells [8]. The excretory-secretory products (ESPs) secreted by *Giardia* could suppress cytokine production in host cells, and concomitantly decrease inflammation to ensure successful colonization [9]. It has been indicated that *Giardia* infection could disrupt the tight junction proteins of IECs [10,11]. *Giardia* stimulation could also result in DNA damage and induce cell cycle arrest at the G1/S boundary in Caco-2 cells, which may be related to increased oxidative stress [12]. Mounting evidence supports the involvement of caspase-dependent IEC apoptosis in pathogenesis of human giardiasis [13,14]. An early study demonstrated that oxidative stress could block the cell cycle of *G. duodenalis* and induce a protease-independent programmed cell death [15]. Reactive oxygen species (ROS)-mediated oxidative stress is implicated in host defense and inflammation, which plays an essential role in controlling and clearing invading pathogens like viruses, bacteria, and parasites [16]. On the other hand, numerous investigations into host–parasite interaction has demonstrated that parasite infection could induce oxidative stress-mediated host cell apoptosis [17,18]. It has been shown that overproduction of ROS is able to cause cell apoptosis through a mitochondria-mediated apoptotic pathway [19]. Our recently published data have indicated that *Giardia* could induce IEC apoptosis through extrinsic pathways [20]. However, it has not yet been determined whether excessive ROS would be produced by *Giardia*-treated cells and be involved in the regulation of host cell apoptosis.

The aim of the present study is to investigate whether *Giardia*-induced Caco-2 cell apoptosis can be regulated by overproduction of ROS in vitro and, if so, to further elucidate the mechanism of ROS-mediated apoptosis. To this end, we measured the effects of trophozoite stimulation on ROS generation and lactate dehydrogenase (LDH) activity of Caco-2 cells, analyzed the changes in mitochondrial morphology and the expression levels of Bax and Bcl-2, the critical regulators of mitochondrial apoptosis pathway, and evaluated the role of ROS in *Giardia*-induced IEC apoptosis.

## 2. Materials and Methods

### 2.1. Cell Culture

The Caco-2 cell line was purchased from Cell Bank of the Chinese Academy of Sciences (Shanghai, China). Cells were maintained in a complete medium consisting of Dulbecco’s Modified Eagle’s Medium (DMEM; Hyclone, Logan, UT, USA), 20% fetal bovine serum (FBS; Cellmax, Beijing, China), 1% penicillin (100 units/mL)/streptomycin (0.1 mg/mL) solution, 1% MEM non-essential amino acid solution (Alphabio, Tianjin, China), and 1% GlutaMAX (Alphabio, Tianjin, China). Cells were passaged at nearly 80% confluency using 0.25% trypsin (Beyotime, Shanghai, China). Only cells from passage 3 to 6 were used.

### 2.2. Parasite Culture

The *G. duodenalis* WB strain typed as assemblage A was obtained from the American Type Culture Collection (Manassas, VA, USA). Trophozoites were cultured in the improved TYI-S-33 culture medium at 37 °C [21]. Before experiments, the original medium was replaced with new cold culture medium to remove dead or unattached trophozoites. The tubes containing trophozoites were placed in ice water and left for 15 min. Localized trophozoites would escape from the tube wall and then be collected by centrifugation for 10 min at 2000 rpm. The collected trophozoites were resuspended in complete culture medium, diluted to the required concentration, and added into the culture medium of growing Caco-2 cells.

### 2.3. LDH Detection

Caco-2 cells were seeded into a 96-well plate in complete medium at 1 × 10^4^ cells per well. The complete medium was replaced with serum-free medium following 12 h incubation. After 3 h of incubation, Caco-2 cells were stimulated with trophozoites at a ratio of 10:1 (trophozoites/cells) for different times (0, 1.5, 3, and 6 h). The maximum LDH was released from the cells treated with 0.1% Triton X-100, which was regarded as positive control. After co-incubation, LDH activity was determined in the supernatant using the LDH assay kit (Beyotime, Shanghai, China), according to the manufacturer’s instructions. The absorbance (Abs) of each well was measured at a wavelength of 490 nm using an enzyme-linked immunosorbent assay (ELISA) reader (BioTek, Winooski, VT, USA).

### 2.4. Apoptosis Analysis

Caco-2 cells (2 × 10^5^ cells/well) were seeded into a 12-well plate in complete medium. The cells were treated for 0, 1.5, 3, and 6 h with trophozoites at a ratio of 10:1 (trophozoites/cells). Then the cells in each well were harvested and stained with propidium iodide (PI) and Annexin V-FITC using an Annexin V-FITC apoptosis detection kit (Beyotime, Shanghai, China). Annexin V possesses a high affinity for phosphatidylserine that is distributed on the surface of apoptotic cells, thereby identifying early or late apoptotic cells. PI was used to detect late apoptotic and necrotic cells. Briefly, the harvested cells were double-stained with Annexin V-FITC and PI, and kept in the dark at room temperature (RT) for 20 min. All experiments were performed on a BD FACS Canto II (BD Biosciences, San Jose, CA, USA). Finally, the rate of apoptosis was determined using the BD FACSDiva software program (BD Pharmingen, San Diego, CA, USA) and the data were analyzed using the Flowjo software program (Tree Star, Ashland, OR, USA).

### 2.5. Cell Cycle Analysis

Caco-2 cells were seeded into a 12-well plate in complete medium at 2 × 10^5^ cells per well. The cells were treated with trophozoites at a ratio of 10:1 (trophozoites/cells) for 0, 1.5, 3, and 6 h. Subsequently, the cells were harvested and fixed in pre-cooled 70% ethanol overnight at 4 °C. The fixed cells were incubated with PI for 30 min at RT while avoiding exposure to the light. The proportion of cells in sub-G0/G1, S, and G2/M phases of cell cycle was determined on a BD FACS Canto II. Data from at least three independent experiments were analyzed using ModFit LT software (Verity Software House, Topsham, ME, USA).

### 2.6. Transmission Electron Microscope (TEM)

The cells with or without *Giardia* trophozoite treatment were harvested by trypsinization and fixed with 2.5% glutaraldehyde overnight at RT. The fixed cells were embedded in Epon-812 resin and then polymerized at 60 °C for 1 h. The sections (55 nm) were cut using a PowerTome XL ultramicrotome (Leica, Buffalo Grove, IL, USA) and then loaded onto a copper grid. Finally, 2% uranyl acetate and lead citrate were used to stain the sections. Mitochondria of trophozoites-treated cells and untreated cells were visualized using a Hitachi H-7650 electron microscope (Tokyo, Japan).

### 2.7. Detections of Intracellular ROS and Glutathione (GSH) Level

Intracellular ROS was measured with the 2′,7′-dichlorofluorescin diacetate (DCFH-DA) that can be converted into fluorescent 2′,7′-dichlorofluorescin (DCF) by cellular peroxides, and the fluorescence of DCF was detected by flow cytometry. Briefly, Caco-2 cells (2 × 10^5^ cells/well) cultured in a 12-well plate were pre-incubated with DMEM containing DCFH-DA (10 μM; Beyotime, Shanghai, China) for 20 min at 37 °C and then washed with phosphate buffer solution (PBS). Cells were treated with *Giardia* at a ratio of 10:1 (trophozoites/cells) for 0, 3, and 6 h, and Rosup was added as a positive control. Fluorescence intensity of DCF was detected using a BD FACS Canto II. Data were analyzed using the BD FACSDiva software program and processed using the Flowjo software.

GSH content was determined via the colorimetric method using a commercial reagent kit (Beyotime, Shanghai, China). At least three measurements were carried out per sample.

### 2.8. Western Blots Analysis

Stimulation of Caco-2 cells with trophozoites at a ratio of 1:10 was performed. Total cellular proteins were prepared by lysing cells in RIPA buffer containing 1% PMSF (Beyotime, Shanghai, China). The enhanced BCA protein assay kit (Beyotime, Shanghai, China) was used to measure the protein concentration. The extracted proteins were separated by SDS-polyacrylamide gels, transferred to a polyvinylidene fluoride membrane, blocked with 5% skim milk, and then incubated with primary antibodies against Bax (ABclonal, Wuhan, China), Bcl-2 (ABclonal, Wuhan, China), cytochrome c (Cyt-c; ABclonal, Wuhan, China), COX IV (ABclonal, Wuhan, China), caspase-9 (CASP9; ABclonal, Wuhan, China), pro-CASP3/cleaved (cl)-CASP3 (ABclonal, Wuhan, China), poly (ADP-ribose) polymerase 1 (PARP; ABclonal, Wuhan, China), and GAPDH (ABclonal, Wuhan, China). Blots were probed with a horseradish peroxidase-linked goat anti-rabbit secondary antibody (ABclonal, Wuhan, China). The proteins were detected and visualized by chemiluminescence (Syngene, Cambridge, UK). The gray values of the protein bands were analyzed using NIH Image J software (Bethesda, MD, USA). Three technical replications were performed for each sample.

### 2.9. Immunofluorescence Assays

Caco-2 cells (5 × 10^4^/well) were seeded into a 24-well plate and then treated with trophozoites (5 × 10^5^/well) for different periods in time (0, 1.5, 3, and 6 h). Then cells were fixed with 4% paraformaldehyde at RT for 30 min, permeabilized with 0.25% Triton-X 100 for 10 min, blocked with 2% bovine serum albumin for 30 min, and incubated with primary antibodies against Bax (1:100), Bcl-2 (1:100), and Cyt-c (1:200) overnight at 4 °C. On the following day, samples were washed with PBS and incubated with FITC-AffiniPure Goat Anti-Rabbit IgG (H + L) (1:200; Jackson, West Grove, PA, USA) in the dark for 1 h at 37 °C. The mitochondria in cells were stained with 200 nM Mito-Tracker® Red CMXRos (Solarbio, Beijing, China) at 37 °C for 30 min and finally the cell nucleuses were counterstained with 2 μg/mL DAPI (Alphabio, Tianjin, China) for 3 min at RT. The fluorescent signal was detected by a Lionheart FX Automated Microscope (BioTek, Winooski, VT, USA). All assays were performed three times in duplicate.

### 2.10. Quantitative Real-Time PCR (qPCR) Analysis

Total RNA was isolated from cells using Trizol reagent (Invitrogen, Carlsbad, CA, USA). Total RNA (1 μg) was reverse transcribed to cDNA using the Hiscript 1st Strand cDNA Synthesis Kit (Vazyme, Nanjing, China). Amplification was performed using SYBR Green qPCR Master Mix (Bimake, Houston, TX, USA) on a LC480 Lightcycler system (Roche, Indianapolis, IN, USA), GAPDH served as an endogenous control. Specific qPCR primers were designed using a NCBI/Primer-BLAST online server (Table 1) and synthesized by SangonBiotech (Shanghai, China). The qPCR data were analyzed using the 2^−ΔΔCt^ method [22].

### 2.11. Mitochondrial Membrane Potential (MMP) Assay

Loss of mitochondrial membrane integrity was measured by using the fluorescent dye JC-1 (Solarbio, Beijing, China), which emits red fluorescence in normal cells with undamaged mitochondria and bright green fluorescence in cells with diminished MMP. The changes in MMP were represented by a ratio of red to green fluorescence. After treatment with *Giardia* trophozoites and 10 μM carbonyl cyanide 3-chlorophenylhydrazone (positive control), cells were stained with JC-1 for 20 min at 37 °C. JC-1 fluorescence was detected using flow cytometry and data were analyzed using a Flowjo software program.

### 2.12. Cell Viability Assay

Caco-2 cells (1 × 10^4^ cells/well) pretreated with or without ROS inhibitor *N*-acetyl-l-cysteine (NAC; 10 mM/well; Abmole Bioscience, Shanghai, China) were stimulated by trophozoites for 6 h. There were also two control groups: NAC- and DMSO-treated. The viability of treated cells was evaluated using a cell counting kit-8 (CCK-8; Apexbio, Houston, TX, USA) assay. The Abs was read at 450 nm on an ELISA reader.

### 2.13. Statistical Analysis

The GraphPad Prism 7.0 was used for data analysis. All results were expressed as means ± standard deviation (SD). Student’s *t*-test (two independent groups) and one-way ANOVA (more than two groups) were used to determine the statistical significance between groups. Differences were considered significant at *p* < 0.05.

## 3. Results

### 3.1. Giardia Trophozoites Promote Apoptosis and Induce Cell Cycle Arrest

**Figure 1:**
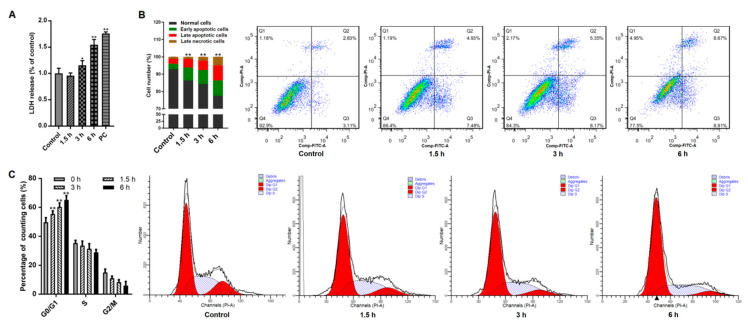
Effects of *Giardia* trophozoite treatment on Caco-2 cell cycle and apoptosis. (**A**) Treatment led to leakage of LDH from Caco-2 cells (n = 5 wells/group). Positive control is abbreviated as “PC”. (**B**) Treatment promoted an increased percentage of apoptotic cells. (**C**) Treatment induced cell cycle arrest at the G0/G1 phase. All results are representative of at least three independent experiments and data are presented as mean ± SD. * *p* < 0.05, ** *p* < 0.01, compared to the control group.

Initially, the influences of *Giardia* trophozoites on Caco-2 cells were measured via evaluating cellular membrane integrity using LDH assay. Under treatment, LDH released from cells was increased time-dependently, suggesting that stimulation damaged cell membrane (Figure 1A). Flow cytometry analysis showed that trophozoites induced cell apoptosis in a time-dependent manner (Figure 1B). The occurrence of cell cycle arrest was evident by observing that the proportion of cells in the G0/G1 phase was significantly increased with the prolongation of treatment time (Figure 1C).

### 3.2. Giardia Trophozoites Trigger ROS Accumulation and Induce Mitochondria Damage in Caco-2 Cells

**Figure 2:**
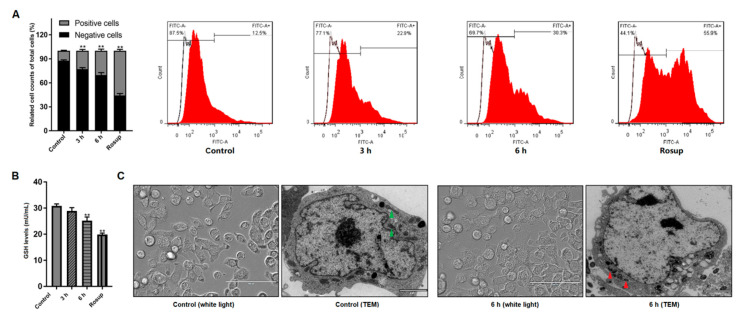
*Giardia* trophozoite treatment induced oxidative stress and mitochondria damage in Caco-2 cells. (**A**) Treatment promoted ROS accumulation. (**B**) Treatment reduced GSH level. (**C**) Treatment resulted in ultrastructural changes of mitochondria (white light, scale bar = 100 μm; TEM, scale bar = 2 μm). All data are representative of at least three independent experiments performed for each group and are shown as mean ± SD. ** *p* < 0.01, compared to the control group.

ROS involves mitochondria-mediated apoptosis induced by parasites as previously noted [23]. Here we examined if ROS level was changed in Giardia-treated cells. Figure 2A illustrated that treatment significantly increased the production of ROS in cells in a time-dependent manner. Meanwhile, the levels of GSH were reduced time-dependently along with the treatment (Figure 2B). Those indicated the presence of Giardia-induced oxidative stress in Caco-2 cells. In TEM observation, compared to the control indicated by green arrows, mitochondrial swelling and disruption of cristae structure were present in Giardia-treated cells (red arrows, Figure 2C).

### 3.3. Giardia Trophozoites Regulate Expression of Bax/Bcl-2 and Localization of Bax in Caco-2 Cells

**Figure 3:**
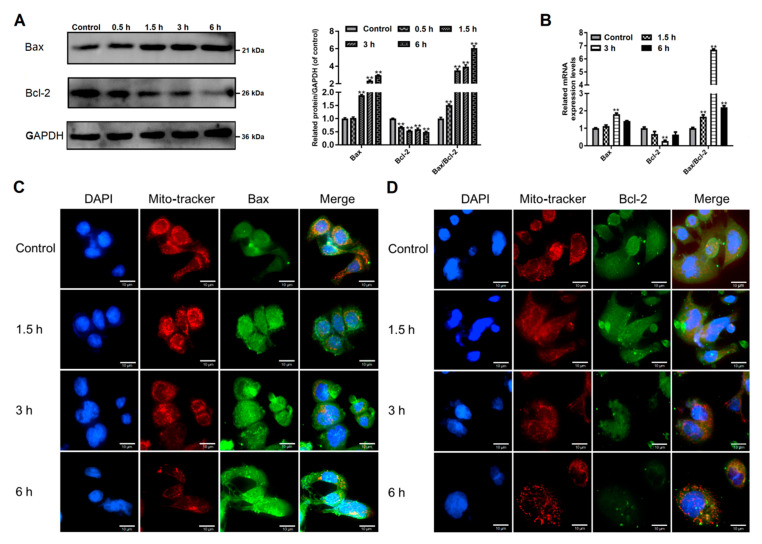
Effects of *Giardia* trophozoite treatment on regulation of Bcl-2 family proteins in Caco-2 cells. (**A**) Treatment up-regulated Bax protein expression and down-regulated Bcl-2 protein expression. (**B**) Treatment increased mRNA expression of Bax and decreased mRNA expression of Bcl-2. (**C**) Treatment promoted translocation of Bax to mitochondria. Scale bar = 10 μm. (**D**) Bcl-2 protein expression was inhibited in trophozoites-treated cells. Scale bar = 10 μm. All data shown are representative of at least three independent experiments and are presented as mean ± SD. ** *p* < 0.01, compared to the control group.

The pro-apoptotic Bax and the anti-apoptotic Bcl-2 belong to the Bcl-2 protein family that controls the mitochondrial apoptotic pathway [24]. Here we observed that trophozoite treatment increased Bax protein expression and decreased Bcl-2 protein expression in Caco-2 cells in a time-dependent manner (Figure 3A). An elevation in a ratio of Bax/Bcl-2 was also observed at the mRNA level, notably at 3 h upon treatment (Figure 3B).

We determined the location of Bax in Caco-2 cells via immunofluorescence assay (Figure 3C). Bax (stains green) is found in the cytoplasm in control cells. Fluorescence intensity of Bax was increased in trophozoites-treated cells. The overlay of red (mitochondria) and enhanced green (Bax) fluorescence implies apoptotic translocation of Bax to mitochondria. Immunofluorescence detection of Bcl-2 (stains green) showed a decreased expression of this protein after trophozoite treatment (Figure 3D). A gradually decreased red fluorescence linked to mitochondria with the time of treatment prolonged indicates the occurrence of mitochondrial damage (Figure 3). From the above findings, we preliminarily concluded that *Giardia*-induced Caco-2 cell apoptosis might be regulated by Bcl-2 family proteins known as the mediators in the mitochondrial apoptotic pathway.

### 3.4. Effects of Giardia Trophozoite Treatment on Mitochondrial Function and Regulation of Apoptotic-Related Proteins in Caco-2 Cells

**Figure 4:**
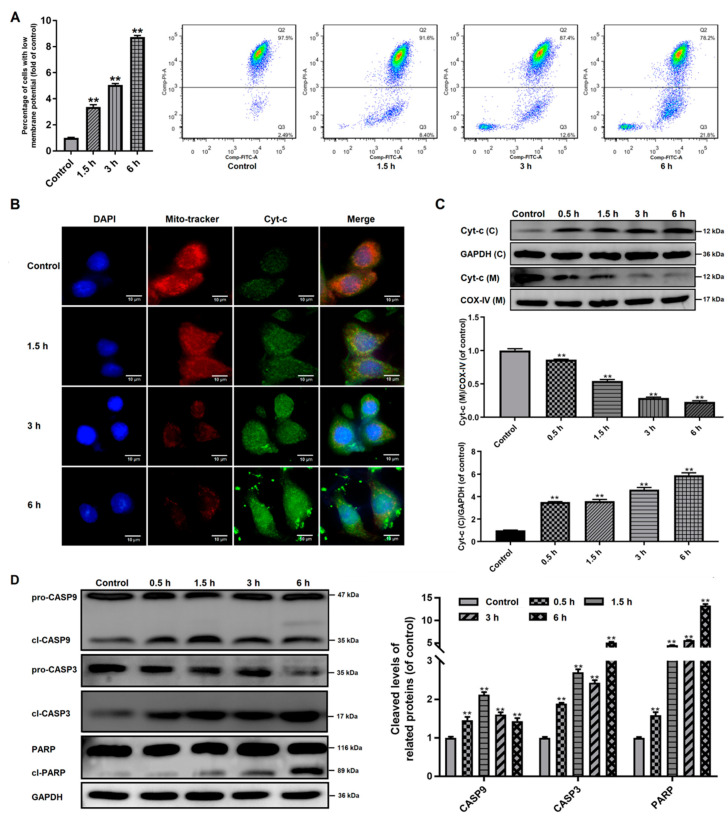
Influences of *Giardia* trophozoite treatment on MMP and regulation of apoptotic signal proteins in Caco-2 cells. (**A**) Treatment induced depolarization of MMP. (**B**,**C**) Treatment induced the release of Cyt-c from the mitochondria into the cytoplasm in the Caco-2 cells. The abbreviations “(C)”and “(M)” mean in cytoplasm and in mitochondria, respectively. GAPDH and COX-IV were used as the internal references for cytoplasm and mitochondria, respectively. (**D**) Treatment induced cleavage of CASP3/9 and PARP. All experiments were repeated at least three times, obtaining similar results. Data are presented as mean ± SD. ** *p* < 0.01, compared to the control group.

It has been shown that Bcl-2 family proteins can induce the mitochondrial outer membrane permeabilization (MOMP) in the apoptosis process [25]. MMP loss is usually understood as an indicator of MOMP and mitochondrial dysfunction [26]. Our study investigated if MOMP occurred in trophozoites-treated cells. As seen in Figure 4A, Caco-2 cells treated with trophozoites induced a time-dependent accumulation of green fluorescence, indicating an MMP loss.

Then we investigated the distribution of Cyt-c in Caco-2 cells, which could be released from the mitochondria into the cytoplasm due to increased MOMP. As shown in Figure 4B, the fluorescence of Cyt-c in Caco-2 cells was increased time-dependently after trophozoite treatment and it was found to gradually accumulate within the cytoplasm. This was also confirmed by western blot analysis (Figure 4C), showing that the protein expression of Cyt-c in the mitochondria was time-dependently decreased, but gradually increased in the cytoplasm.

CASP9 has been reported to be activated by the apoptosome-like Cyt-c in cytoplasm, which served as an initiator caspase in the intrinsic or mitochondrial pathway [27]. Western blot analysis showed that trophozoite treatment could significantly induce the activation of CASP9 and its downstream effector CASP3 in Caco-2 cells (Figure 4D). Additionally, exposure of Caco-2 cells to trophozoites could also result in cleavage of PARP known as a marker of apoptosis (Figure 4D).

### 3.5. The Role of ROS Production in Giardia Trophozoites-Induced Caco-2 Cell Apoptosis

**Figure 5:**
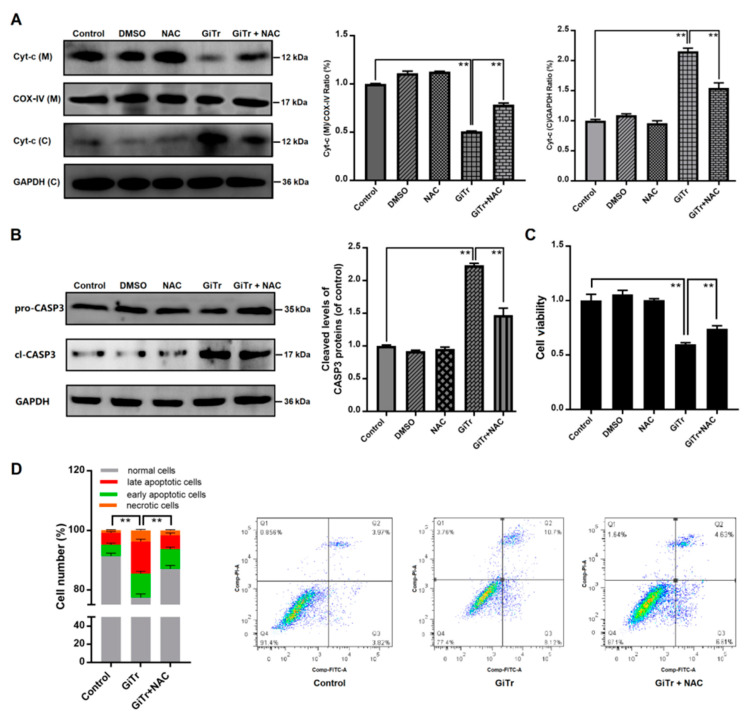
The role of ROS production in regulation of Caco-2 cell apoptosis induced by *Giardia* trophozoites. Cells pre-incubated with or without NAC for 3 h were then treated with trophozoites for 6 h. (**A**) The release of Cyt-c from mitochondria into cytoplasm was inhibited by NAC in trophozoites-treated cells. The abbreviations “(C)” and “(M)” represent in cytoplasm and in mitochondria, respectively. COX-IV and GAPDH were used as mitochondrial and cytosolic internal controls, respectively. (**B**) NAC inhibited trophozoites-activated CASP3. (**C**) NAC increased the viability of trophozoites-treated cells (n = 5 wells/group). (**D**) NAC inhibited trophozoites-induced cell apoptosis. All the data are representative of at least three experiments yielding similar results and are presented as the mean ± SD. *Giardia* trophozoite is abbreviated as “GiTr”. ** *p* < 0.01, compared to the control group.

The intracellular ROS level was increased during trophozoite treatment as mentioned earlier (Figure 2A). We used NAC to inhibit ROS generation to analyze the role of ROS in regulation of trophozoites-induced apoptosis. It was shown in Figure 5A that pre-incubation of Caco-2 cells with NAC for 3 h before exposure to trophozoites could block the release of Cyt-c. The activation of CASP3 was diminished by NAC in trophozoites-treated cells (*p* < 0.01, Figure 5B). In CCK-8 analysis, cell viability was significantly decreased (*p* < 0.01) at 6 h post-infection and this effect could be significantly inhibited by NAC (*p* < 0.01, Figure 5C). In addition, it was indicated that NAC could decrease apoptosis of Caco-2 cells induced by trophozoites (*p* < 0.01, Figure 5D).

## 4. Discussion

**Figure 6:**
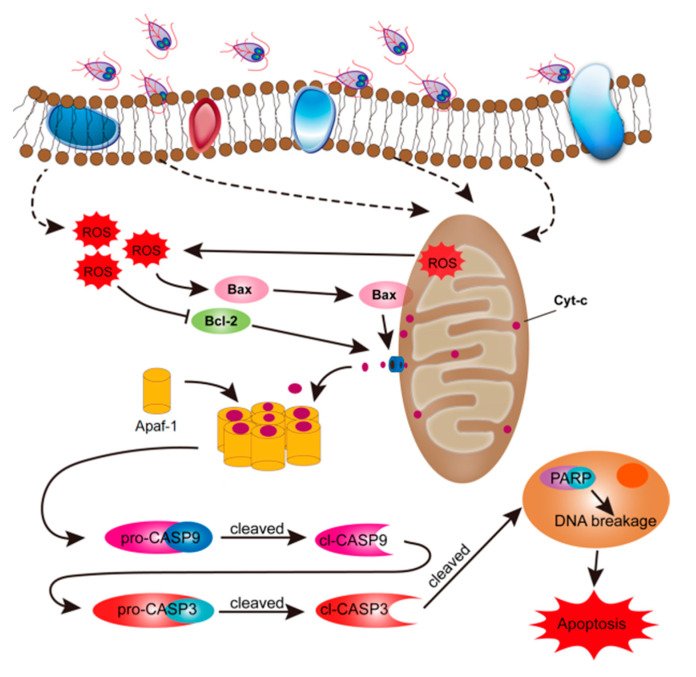
Schematic figure of the molecular mechanism by which *Giardia* trophozoites induce apoptosis in Caco-2 cells. The role of ROS in trophozoites-induced cell apoptosis was assessed. Briefly, trophozoite stimulation may preferentially induce intracellular ROS production in Caco-2 cells, which caused Cyt-c release from the mitochondria and apoptotic executioner CASP3 activation. Activated CASP3 led to cleavage of PARP and initiated apoptosis in Caco-2 cells.

In this study, we analyzed whether *G. duodenalis*-induced cell apoptosis can be mediated by ROS. The results demonstrated that trophozoites promoted generation of intracellular ROS in Caco-2 cells. Overproduction of ROS led to an increase in the ratio of Bax to Bcl-2 protein expression, MOMP, Cyt-c release into the cytoplasm, activation of CASP3/9, and finally occurrence of cell apoptosis. These data suggested that ROS production was involved in the process of trophozoites-induced Caco-2 cell apoptosis via modulating the mitochondrial apoptotic pathway (Figure 6).

*G. duodenalis* assemblage A has been widely studied since it is a commonly identified genotype in human giardiasis [28]. Our recent research showed that *Giardia* trophozoites could induce Caco-2 cell apoptosis via the extrinsic pathway initiated by activation of the death receptor, tumor necrosis factor receptor type 1 (TNFR1) [20]. However, TNFR1 might not be the sole factor responsible for *Giardia* infection and the apoptotic process. Oxidative stress was shown to be capable of inducing a caspase-independent programmed cell death in *G. duodenalis* [15]. Oxidative stress mediated by intracellular ROS has been reported in association with the intrinsic pathway of cell apoptosis [29,30]. However, it is still uncertain whether excessive generation of ROS plays a role in anti-*Giardia* battle and promotion of intrinsic IEC apoptosis. As expected, here we found that excessive ROS could be produced by Caco-2 cells in response to *Giardia* stimulation in vitro. The increased release of LDH is indicative of Caco-2 cell membrane disruption, which may contribute to ROS release outside to defend *Giardia* infection. We also demonstrated that trophozoite stimulation could induce Caco-2 cell apoptosis and cell cycle arrest in the G0/G1 phase. IEC apoptosis has been linked with pathogenesis of diarrhea and inflammatory bowel disease (IBD) [31,32], which might be answerable for the typical gastrointestinal symptoms of human giardiasis. In addition, it has been suggested that IBD might be closely associated with oxidative stress derived from overproduction of ROS [33]. Mitochondria are considered the main intracellular source of ROS, and excessive generation of ROS can result in cell apoptosis [34,35]. Herein, the mitochondria in *Giardia*-treated cells were swollen with irregular shape, and the mitochondrial cristae were fragmented and disappeared. Structural damage to mitochondria was considered an important cause of respiratory function disorders that can result in generation of inadequate ATP and excessive ROS and cause negative impacts on the life activities of cells [36]. Furthermore, we observed a significant reduction of GSH content in Caco-2 cells stimulated by *Giardia*, clearly signifying the existence of oxidative stress.

It was known that ROS-caused oxidative damage could elevate the Bax/Bcl-2 ratio, induce the opening of mitochondrial permeability transition pores, cause a decline in MMP, and promote the release of Cyt-c from mitochondria into the cytoplasm [37]. These changes were also observed in *Giardia* trophozoites-treated cells in this study. Cyt-c, a member of class 1 of the c-type cytochrome family, can perform different biological functions depending on its localization [38]. It was indicated that increased MOMP could directly result in Cyt-c release from mitochondria [39,40]. Once being released into the cytoplasm, Cyt-c can recruit pro-CASP9 to form the apoptosome and then the apoptotic initiator caspase, CASP9, was activated [27]. CASP9 can cleave and activate the apoptotic effector CASP3, and then PARP, a well-known substrate of CASP3, was cleaved and activated by cl-CASP3 [41]. In our study, there was a significant increase in the levels of cl-CASP9, cl-CASP3, and cl-PARP in *Giardia*-treated Caco-2 cells. Similarly, host cell apoptosis can also be induced by another flagellate, *Trichomonas vaginalis*, via activation of CASP3 and PARP [23]. Moreover, pretreatment of *Giardia*-treated cells with NAC could restrain Cyt-c release, CASP3 activation, and mitochondrial apoptosis, suggesting an important role of ROS in the process of regulation. Despite the advances made so far, the mechanism of IEC death caused by *Giardia* infection needs further investigation.

*Giardia* is a surface-colonizing noninvasive pathogen, the role of its specific virulence factors in mediating host–parasite interactions remains to be fully elucidated, notably those concerning host cell death. In the intrinsic pathway, a basic event resulting in caspase activation is the mitochondrial membrane permeability that can be increased by a number of internal stimuli [42]. It could thus be speculated that, during *Giardia* infection, transfer of some virulence proteins into the host cells might occur to initiate the mitochondrial pathway of apoptosis. A recent review has addressed the effects of ESPs released from *Giardia* on host cell signaling and immune regulation [43]. ESPs have also been shown to function during the trophozoite attachment process [44,45]. Another piece of research confirmed the presence of ESPs in Caco-2 cells and indicated that the internalized ESPs could suppress inflammatory response via regulating NF-κB- and MAPK-signaling pathways [9]. In addition, the *Giardia*-secreted sphingolipid ceramide-1-phospate was depicted in association with IBD during acute infection [46]. Our future work will identify the molecular players, potentially ESPs, mediating *Giardia*-associated host cell death signaling and attempt to understand how they work.

In conclusion, our study exhibited that *G. duodenalis* could induce Caco-2 cell apoptosis via ROS-mediated mitochondrial pathway. It has been known that IEC apoptosis caused by *Giardia* attachment could disrupt epithelial barrier functions and promote renewal of the intestinal epithelium, which might drive the development of giardiasis [13]. Our findings provided some new insights into the understanding of the pathogenicity mechanisms of *Giardia*, as well as the development of new strategies for the control and treatment of giardiasis.

## Figures and Tables

**Table 1:** The primer pairs used for qPCR analysis.

Gene	Accession No.	Primer (5′ to 3′)	Product Size
Bax	NM_138763.4	Forward: CCCGAGAGGTCTTTTTCCGAG, Reverse: CCAGCCCATGATGGTTCTGAT	155 bp
Bcl-2	XM_017025917.2	Forward: GGTGGGGTCATGTGTGTGG, Reverse: CGGTTCAGGTACTCAGTCATCC	89 bp
GAPDH	NM_001357943.2	Forward: ACAACTTTGGTATCGTGGAAGG, Reverse: GCCATCACGCCACAGTTTC	101 bp
